# Transcranial direct current stimulation in attention-deficit hyperactivity disorder: A meta-analysis of neuropsychological deficits

**DOI:** 10.1371/journal.pone.0215095

**Published:** 2019-04-12

**Authors:** Mohammad Ali Salehinejad, Miles Wischnewski, Vahid Nejati, Carmelo M. Vicario, Michael A. Nitsche

**Affiliations:** 1 Ruhr-University Bochum, International Graduate School of Neuroscience, Bochum, Germany; 2 Leibniz Research Centre for Working Environment and Human Factors, Department of Psychology and Neurosciences, Dortmund, Germany; 3 Donders Institute for Brain, Cognition, and Behaviour, Radboud University Nijmegen, The Netherlands; 4 Department of Psychology, Shahid Beheshti University, Tehran, Iran; 5 Department of Psychology, University of Regensburg, Regensburg, Germany; 6 University of Messina, Department of Scienze Cognitive della Formazione e degli Studi Culturali, Messina, Italy; 7 University Medical Hospital Bergmannsheil, Department of Neurology, Bochum, Germany; University of Bologna, ITALY

## Abstract

Transcranial direct current stimulation (tDCS) is a promising method for altering cortical excitability with clinical implications in neuropsychiatric diseases. Its application in neurodevelopmental disorders especially attention-deficit hyperactivity disorder (ADHD), is in early stage and promising but its effectiveness has not been systematically examined yet. We conducted a meta-analysis on the effectiveness of tDCS on the most studied neuropsychological symptoms of ADHD, which is the first reported meta-analysis of tDCS studies on ADHD. Data from 10 randomized controlled studies (including 11 separate experiments) targeting inhibitory control, and/or working memory (WM) in ADHD were included. Results show that overall tDCS significantly improved inhibitory control. Sub-analyses further show that dorsolateral prefrontal cortex (dlPFC) (but not right inferior frontal gyrus) tDCS and anodal (but not cathodal) tDCS significantly improved inhibitory control with a small effect size. Anodal dlPFC-tDCS had the largest significant effect on inhibitory control with a small-to-medium effect size. Additionally, a significant improving effect of tDCS on inhibitory control accuracy (but not response time) and WM speed (but not accuracy) were found. Overall, this meta-analysis supports a beneficial effect of tDCS on inhibitory control and WM in ADHD with a small-to-medium effect size. TDCS seems to be a promising method for improving neuropsychological and cognitive deficits in ADHD. However, there might be a dissociation between neuropsychological deficits and clinical symptoms of ADHD and therefore, the significance of this meta-analysis for clinical purposes is limited. Future studies should systematically evaluate the role of inter-individual factors (i.e., ADHD subtype, types of the deficit) and stimulation parameters (i.e., site, polarity, intensity, duration, repetition rate) on tDCS efficacy in ADHD population and examine whether benefits are long-term.

## Introduction

Attention-deficit hyperactivity disorder (ADHD) is the most common neurodevelopmental disorder with an estimate of 11% prevalence in school-age children [1]. ADHD is primarily characterized by symptoms of inattention, hyperactivity, impulsivity [2] and various cognitive dysfunctions [3] that often persist into adolescence and adulthood. Apart from symptoms of hyperactivity and inattentiveness, a wide range of cognitive deficits is observed in individuals with ADHD such as problems in attention, inhibitory control working memory (WM), planning, problem-solving and executive functions [4–7]. Executive dysfunctions, especially inhibitory control and working memory, are pervasive and influential in ADHD pathophysiology to the extent that ADHD was labeled a disorder of cognitive control [7, 8] but, cognitive deficits in ADHD are heterogeneous [9–11].

A precise description of ADHD neuropathology is difficult to delineate due to its neuropsychological heterogeneity [12, 13], and substantial overlap between ADHD and typically developing children [14]. However, based on neuroimaging and neuropsychological findings relatively distinct brain regions can be identified to account for ADHD hallmark symptoms. Poor inhibitory control resulting from executive dysfunctions (i.e., inhibition-based model) and impulse-control deficits leading to hyperactivity (i.e., motivational dysfunction model) are two influential theories for neural foundations of ADHD [15, 16]. These major symptoms have been linked to frontostriatal circuitry and prefrontal cortex (PFC) abnormalities and can be diverged in the “prefrontal hypothesis of ADHD” [17]. According to this hypothesis, prefrontal regions including the dorsolateral PFC (dlPFC), orbitofrontal cortex (OFC) and inferior frontal gyrus (IFG) have altered activity in ADHD. Specifically, hypoactivity of the bilateral dlPFC, right inferior frontal gyrus (rIFG) and OFC, as well as smaller prefrontal volumes, are associated with behavioral deficits in ADHD [18–22]. Additionally, an adaptive increase of posterior parietal activity accompanies the respective hypoactivity of frontostriatal regions during performance of executive tasks [19]. These findings suggest that ADHD symptoms/causes stem from disturbances in large-scale brain networks [23, 24] and structural, functional and neurochemical abnormalities in cortical and subcortical structures [13, 17, 25], which should be considered in therapeutic interventions.

Pharmacological and non-pharmacological interventions have been among the most commonly used and recommended treatment options in ADHD. Pharmacological interventions can broadly be divided into stimulant and non-stimulant medications [26, 27]. Non-pharmacological or psychological interventions can be categorized into behavioral interventions, cognitive training and neurofeedback training [27, 28]. Stimulant medications have been so far the most effective psychopharmacological treatment and adherence to treatment is reported relatively high. Although current treatment options especially pharmacological interventions have been relatively effective and successful, the efficacy of these treatment options has been inconsistent in ADHD [27]. For example, the efficacy of pharmacological treatments is not long-lasting enough [29] and suffers from the partial response or non-response [30], adverse effects and relatively poor adherence [31]. On the other hand, neurofeedback training is found to have limited effects on ADHD when rated by blind evaluators [32]. Due to the heterogeneity of ADHD pathophysiology, symptoms and treatment response, there might be a need for new treatment or complementary treatment options, especially regarding interventions based on recent findings of the neuropathology of ADHD.

The significant role of brain abnormalities in ADHD symptomology encouraged researchers to probe new treatment options that target ADHD symptoms by modulating, altering and remediating deficient neurocognitive brain functions. Transcranial direct current stimulation (tDCS) is a non-invasive, painless, and well-tolerated brain stimulation technique that has been gaining increased popularity in human neuroscience research in both healthy and clinical populations during last decade [33–35]. It induces a weak direct current applied to the scalp which modulates cortical excitability by shifting resting membrane potential, [36] and can induce both, acute and neuroplastic alterations of cortical excitability at the macroscopic level [37]. In the motor cortex, it has been suggested that anodal stimulation increases cortical excitability while cathodal stimulation decreases it [38] although such polarity-dependent effect is not always linear and could be affected by external and individual difference factors especially stimulation parameters such as current density, polarity, stimulation duration and/ or geometrical montage of electrodes [39, 40].

TDCS has been applied for modulating a variety of cognitive functions [41–44] or improving symptoms/deficits in healthy and clinical populations [45] (for an overview see [46, 47]). Its application in neurodevelopmental populations, especially ADHD, has gained attention in recent years [48, 49] while the efficacy of the method in ADHD is still unclear and needs further research. Different stimulation parameters and montages have been used in previous studies and different symptoms and deficits have been targeted by tDCS which does not evaluate the potential of this method in ADHD. Moreover, most clinical tDCS studies so far have focused on adult populations and there is a great need for more research into their therapeutic uses in pediatric patients [49, 50]. Considering the fact that pediatric brains still have to go through various stages of neurodevelopment and have accelerated neuroplasticity compared to adults [51], tDCS could be considered a potential therapy for some pediatric disorders, particularly when there are no other safe viable alternatives [52]. That being said, knowledge about tDCS effects in ADHD, is still relatively limited and warrants further investigation especially through systematic review or meta-analytic studies.

To date, meta-analysis or specific systematic review is available for the effects of tDCS on ADHD. The present meta-analysis, therefore, aims to investigate the effectiveness of tDCS on neuropsychological deficits in ADHD. Moreover, we did further sub-analyses on the available studies in order to (1) evaluate overall and deficit-specific efficacy of tDCS in ADHD, (2) identify the brain regions that are prominently involved in ADHD pathophysiology tDCS response, and (3) examine stimulation parameters that are important for tDCS efficacy in ADHD. Since inhibitory control and WM deficiencies are among the major neuropsychological deficits of ADHD, [53] and mediated by the genetic risk for ADHD, [54], we performed a meta-analysis on the effects of tDCS on inhibitory control and WM performance in respective patients.

## Method

Our meta-analysis follows the guidelines of the Preferred Reporting Items for Systematic reviews and Meta-Analyses (PRISMA) [55] which consists of a checklist intended to facilitate preparation and reporting review/meta-analysis studies by identifying, selecting, and critically appraising relevant research, and collecting and analyzing data from the included studies. A brief version of the PRISMA checklist is available as supporting information (See S1 Table). The following steps are based on the PRISMA protocol.

### Eligibility criteria

To ensure a high level of methodological accuracy, only peer-reviewed published studies were included in our analysis. Only studies on humans using tDCS in experimental designs with the control condition (i.e., sham stimulation and/or baseline control) with reported full-text in English were retained. Studies were included if (1) they had a randomized placebo- (sham^50^) controlled or baseline-controlled design, (2) measured at least inhibitory control, and/or working memory performance, (3) reported data (text, figures, tables, appendices) which contained effect size values or mean and standard deviation values in order to calculate effect sizes. Participants in the studies were required to be between 3 to 18 years of age (for childhood and adolescence ADHD) and 18 to 65 years of age (for adult ADHD) and to have a clinical diagnosis of ADHD (any subtype) based on the Diagnostic and Statistical Manual of Mental Disorders (DSM) or meet accepted cut-offs on validated ADHD symptom rating scales. Studies which included patients under medication for ADHD treatment in either the control or the active arm were not excluded.

### Search strategy and study selection

**Fig 1:**
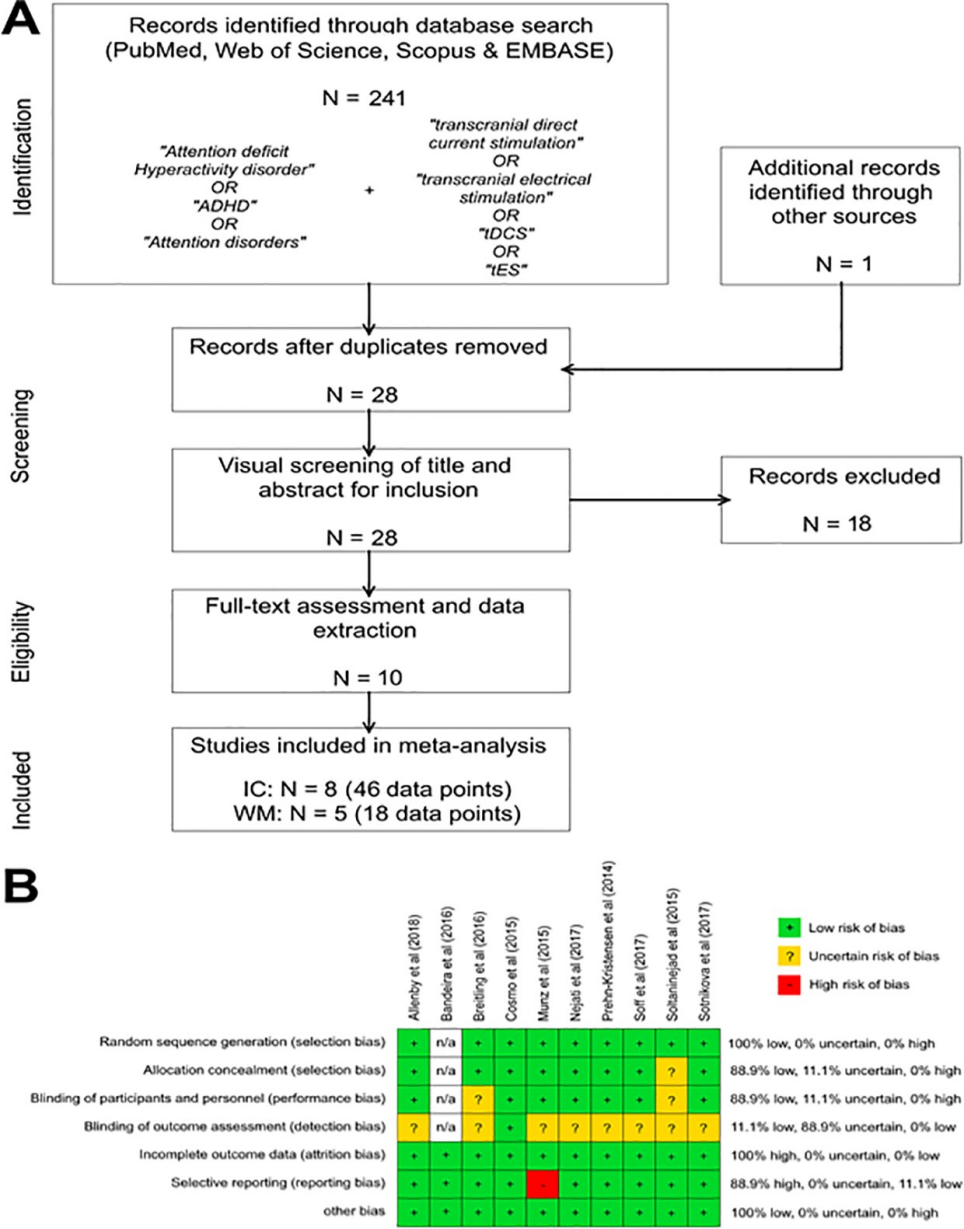
(A) Preferred Reporting Items for Systematic Reviews and Meta-Analyses (PRISMA) flow diagram of selection of studies, (B) Bias assessment in individual studies. tDCS = Transcranial Direct Current Stimulation; ADHD = attention-deficit hyperactivity disorder; IC = Inhibitory Control; WM = Working Memory.

A comprehensive literature search was conducted, including the PubMed database, Web of Science, Medline, and Scopus with the final search updated on January 2019. Search terms included attention-deficit hyperactivity disorder OR ADHD OR attention disorders AND transcranial direct current stimulation OR tDCS OR transcranial electrical stimulation OR tES (see Fig 1). Furthermore, a manual search was carried out over the reference sections of retrieved studies and review articles. After removal of duplicates, the full text was assessed and studies that were eligible according to the above-mentioned inclusion criteria were selected. The final search identified a total of 241 articles with 1 additional article identified by reference lists and recent reviews. Records were reduced to 28 articles after removing duplications. A content expert examined each title and abstract for eligibility. 18 articles were excluded for not meeting the inclusion criteria. Thus 10 articles remained for full-text assessment and data extraction. 8 of these studies investigated inhibitory control and 5 studies explored tDCS effects on WM performance. Articles were screened and selected independently by the first and second authors. Respective study characteristics are summarized in Tables 1 and 2.

**Table 1:** Characteristic of studies included in meta-analysis for the effecs of tDCS on inhibitory control.

#	Authors	N	Mean age	tDCS montage (target/reference)	Intensity	Duration	Polarity	On-/off-line	Control	Task	Outcome	Hedges’ g
1	Allenby et al (2018)	37	37.17 (range 18–56)	F3/Fp2 (25 cm^2^ both)	2 mA	3 days x 20 min	Anodal	Offline	Baseline + sham	CPT	False positive errors	0.42
											True positive errors	-0.06
											Response time	-0.11
				F3/Fp2 (25 cm^2^ both)	2 mA	3 days x 20 min	Anodal	Offline	Baseline + sham	SST	Reaction time	-0.18
2	Bandeira et al (2016)	9	11.1 ± 2.8	F3/Fp2 (35 cm^2^ both)	2 mA	5 days x 30 min	Anodal	Offline	Baseline	NEPSY II	Total errors	0.12
											Completion time	0.54
3	Breitling et al (2016)	21	14.33 (range 13–17)	F8/mastoid (35 cm^2^ both)	1 mA	20 min	Anodal	Online	Sham	Flanker task	Omission errors	-0.11
											Comission errors	0.46
											Reaction time	-0.14
											Reaction time variability	0.13
				F8/mastoid (35 cm^2^ both)	1 mA	20 min	Cathodal	Online	Sham	Flanker task	Omission errors	-0.60
											Comission errors	0.17
											Reaction time	0.13
											Reaction time variability	-0.02
4	Cosmo et al (2015)	30	31.8 ± 11.6	F3/F4 (35 cm^2^ both)	1 mA	20 min	Anodal	Offline	Sham	Go/No-go task (letters)	Correct responses	-0.39
											Omission errors	1.17
											Comission errors	-0.15
										Go/No-go task (fruits)	Correct responses	1.09
											Omission errors	0.44
											Comission errors	0.24
5	Munz et al (2015)	14	12.3 ± 1.4	F3+F4/both mastoids (0.5 cm^2^ all)	0–0.25 mA (oscillatory)	5 x 5 min	Anodal	Offline	Sham	Go/No-go task	Reaction time	0.88
											Reaction time variability	0.83
6	Nejati et al (2017) experiment 1	15	10 ± 2.2	F3/F4 (25 cm^2^ both)	1 mA	15 min	Anodal	Offline	Sham	Go/No-go task	Go accuracy	0.14
											No-go accuracy	0.56
											Reaction time	-0.41
										Stroop task	Accuracy	1.11
											Reaction time	0.98
7	Nejati et al (2017) experiment 2	10	9 ± 1.8	F3/Fp2 (25 cm^2^ both)	1 mA	15 min	Anodal	Offline	Sham	Go/No-go task	Go accuracy	0.41
											No-go accuracy	0.57
											Reaction time	-0.20
				F3/Fp2 (25 cm^2^ both)	1 mA	15 min	Cathodal	Offline	Sham	Go/No-go task	Go Accuracy	0.41
											No-go accuracy	1.04
											Reaction time	-0.20
8	Soltaninejad et al (2015)	20	Range 15–17	F3/Fp2 (35 cm^2^ both)	1.5 mA	8 min	Anodal	Offline	Sham	Go/No-go task	Go accuracy	-0.05
											No-go accuracy	0.03
											Reaction time	0.23
										Stroop task	Accuracy	0.57
											Reaction time	0.23
				F3/Fp2 (35 cm^2^ both)	1.5 mA	8 min	Cathodal	Offline	Sham	Go/No-go task	Go accuracy	-0.54
											No-go accuracy	0.73
											Reaction time	-0.02
										Stroop task	Accuracy	0.33
											Reaction time	0.11
											Reaction time	0.02
9	Sotnikova et al (2017)	13	14.33 ± 1.3	F3 (13 cm^2^)/ Cz (35 cm^2^)	1 mA	30 min	Anodal	Online	Sham	Go/No-go	accuracy (hits+correct rejections/total number of stimuli)	-1.03
											Reaction time	0.16
											Reaction time variability	-0.14

tDCS = transcranial direct current stimulation; F3 = left dlPFC; F4 = right dlPFC; F8 = inferior frontal gyrus; Fp1 = left supraorbital area; Fp2 = right supraorbital area; online = task performance during tDCS; offline = task performance after tDCS; CPT = Conners Continuous Performance Task; SST = Stop Signal Task (SST).

**Table 2:** Characteristic of studies included in the meta-analysis for the effects of tDCS on working memory.

#	Authors	N	Mean age	tDCS montage (target/reference)	Intensity	Duration	Polarity	On-/off-line	Control	Task	Outcome	Hedges’ g
1	Bandeira et al (2016)	9	11.1 ± 2.8	F3/Fp2 (35 cm^2^ both)	2 mA	5 days x 30 min	Anodal	Offline	Baseline	Digit span forward	Amount	-0.87
										Digit span backward	Amount	-0.40
										Corsi cube forward	Amount	-0.45
										Corsi cube backward	Amount	0.08
2	Nejati et al (2017) experiment 1	15	10 ± 2.2	F3/F4 (25 cm^2^ both)	1 mA	15 min	Anodal	Offline	Sham	1-back task	Accuracy	0.15
											Reaction time	1.70
3	Nejati et al (2017) experiment 2	10	9 ± 1.8	F3/Fp2 (25 cm^2^ both)	1 mA	15 min	Anodal	Offline	Sham	1-back task	Accuracy	1.14
											Reaction time	0.82
				F3/Fp2 (25 cm^2^ both)	1 mA	15 min	Cathodal	Offline	Sham	1-back task	Accuracy	0.53
											Reaction time	0.52
4	Prehn-Kristensen et al (2014)	12	12.1 (range 10–14)	F3+F4/both mastoids (0.5 cm^2^ all)	0–0.25 mA (oscillatory)	5 x 5 min	Anodal	Offline	Baseline + sham	Digit span	Amount	-0.61
5	Soff et al (2017)	15	14.2 ± 1.2	F3 (3.14 cm^2^)/Cz (12.5 cm^2^)	1 mA	5 days x 20 min	Anodal	Offline	Baseline + sham	QB (1-back) task	QB score (errors and reaction time)	0.50
6	Sotnikova et al (2017)	13	14.33 ± 1.3	F3 (13 cm^2^)/ Cz (35 cm^2^)	1 mA	30 min	Anodal	Online	Sham	1-back task	Accuracy	-0.99
											Reaction time	-0.05
											Reaction time variability	0.18
										2-back task	Accuracy	-1.14
											Reaction time	0.65
											Reaction time variability	1.06

tDCS = transcranial direct current stimulation; F3 = left dlPFC; F4 = right dlPFC; Fp2 = right supraorbital area; online = task performance during tDCS; offline = task performance after tDCS; QbTest = Quantified Behavior Test.

### Outcome variables

We limited our focus on neuropsychological variables that were targeted in most ADHD studies using tDCS. Based on the most commonly available data, inhibitory control and WM were identified as the variables for which the data were extracted. Inhibitory control and WM are central components of ADHD executive dysfunctions, as proposed by causal models of ADHD, [14, 53, 56] and encompass the most consistently impaired domains in ADHD [8]. Moreover, inhibitory control and WM are major neuropsychological deficits from the neuroscientific perspective, including the prefrontal-striatal model of ADHD [57]. For the meta-analysis on inhibitory control, the following tasks were included: (1) Go/No-Go, (2) Stop Signal Task (SST), 3) Flanker, 4) Stroop, 5) Continuous Performance Test (CPT), and 6) Neuropsychological Development Assessment (NEPSY II). In the Go/No-Go task, inhibitory process is reflected by the ability to inhibit a motor action in the case of specific number of “No-Go” trial [58]. Similarly, in the SST, participants should stop responding to “Go” trials after the stop signal is presented which shows the ability to inhibitory control. In the Stroop task, inhibitory control is reflected by the ability to suppress the meaning of a written word and focus on the color [59]. For the Flanker task response, inhibition is measured by identifying the target defined by its location while ignoring one or more distracting items that flank the targets in the same or opposite direction [60]. In the CPT, which is primarily a test of attention, the errors of commission and omission reflect inhibitory control ability [61]. Finally, in NEPSY-II inhibitory control is measured by looking at a series of shapes or arrows and naming the shape or direction or an alternative response, depending on the color or shape of the arrow [62]. For the meta-analysis on WM, the following tasks were included: 1) N-back, 2) Digit Span, and 3) Corsi block-tapping test. In the N-back task, subjects need to identify a stimulus that repeats the one presented “n” items before its onset. In the digit span task, subjects are read or shown a list of digits and asked to recall them in order. In the Corbi Cubes test, the subject repeats sequences of touches in different cubes (either forward or backward) [63].

### Risk of assessment bias

Quality assessment of the included studies was performed using the Cochrane Collaboration’s tool for risk of bias in randomized trials [64]. For each study, the authors judged the risk of selection bias, performance bias, detection bias, attrition bias, reporting bias, and other biases. Risk of bias was categorized as low, high or uncertain. The final rating was established through consensus with the involvement of the senior author. The assessment of each study and the percentage of bias are presented in Fig 1B.

### Data extraction and statistical analysis

Hedges’ *g* was used as a measure of effect size. This effect size reflects the tDCS-induced difference compared to 1) sham, 2) baseline or 3) sham and baseline (i.e. posttest–pretest tDCS vs posttest–pretest sham). When the effect size Cohen’s *d* was reported, the effect sizes were extracted directly from the selected articles. Otherwise, *d* was calculated using means and pooled standard error of mean, which were gathered from the results section, figures or tables. From these effect sizes, Hedges’ *g* values were calculated to correct for effect inflation due to small sample sizes [65]. In this meta-analysis, positive values reflect a tDCS-induced increase of inhibitory control or WM performance, whereas negative values indicate a tDCS-induced decrease of inhibitory control or WM performance compared to sham/baseline values. A weighted average was calculated from these Hedges’ *g* values to determine the cumulative effect size (Ē) and 95% confidence intervals. Subsequently, the Z-statistic and p-value were calculated to investigate whether Ē differed significantly from zero.

For the main investigation on the effects of tDCS on inhibitory control in ADHD patients, three meta-analytic steps were performed. First, an unsigned analysis, in which the general effect of frontal tDCS, independent of polarity was analyzed. This analysis included 46 extracted effect size values. We further explored the effect of electrode montage by comparing tDCS setups that primarily targeted the dlPFC (38 extracted effect size values) or the rIFG (8 extracted effect size values). Second, a signed analysis was performed in which the effects of anodal (34 extracted effect size values) and cathodal tDCS (12 extracted effect size values) were determined separately. This analysis was then followed by an exploration of tDCS montage. Finally, since the majority of outcome measurements investigated both, accuracy (i.e., amount of correct responses or error rate) and speed (i.e. reaction time or reaction time variability) on inhibitory control tasks, separate analyses were performed for these variables (27 and 19 extracted effect size values respectively). For the WM analyses, not enough observations could be gathered for cathodal tDCS stimulation (n = 2) and therefore no separate analyses were performed.

For all analyses, the sample distribution was checked for normality by the Kolgomorov-Smirnov test. Additionally, total heterogeneity (Qtotal) was tested against the χ^2^ distribution with n-1 degrees of freedom to determine whether the variance of effect sizes was greater than to be expected from sampling error [65]. To further investigate possible effects of publication bias, the fail-safe number, based on the Rosenthal method (α < 0.05), was calculated, which gives an indication of the amount of null findings that are needed to render the cumulative effect non-significant [66]. Data were analyzed using MetaWin 2.1[67] and IBM SPSS 22.0. All statistical tests were tested against a significance level of α ≤ 0.05 (two-tailed).

## Results

### Overview

**Fig 2:**
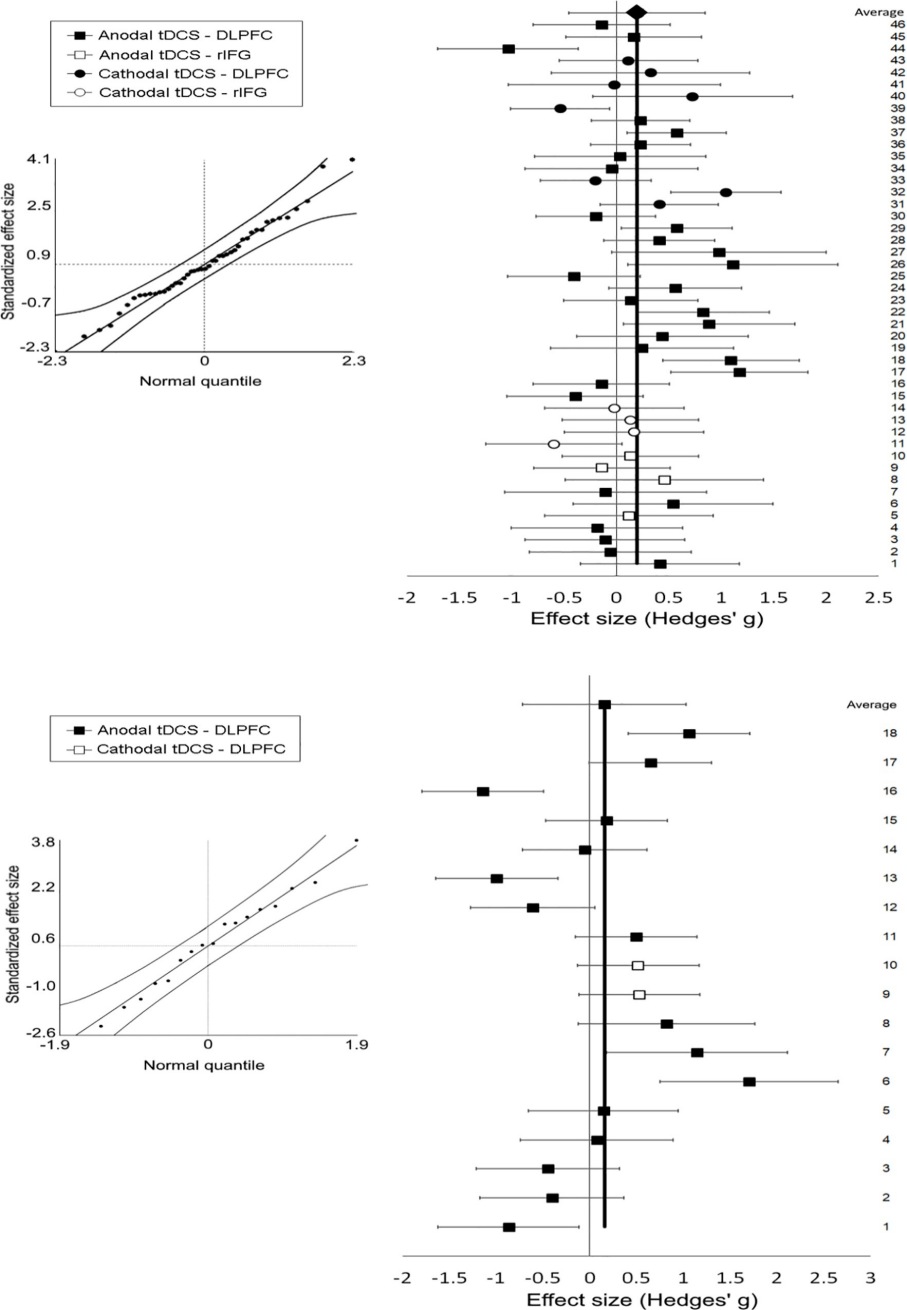
Meta-analysis and forest plot results including Hedges’ g and 95% confidence interval and Cumulative effect size of tDCS on inhibitory control (top) and working memory (down).

In total, 11 separate experiments from 10 studies published from 2014—April 2018 were included in this meta-analysis. In 8 of 10 studies (or 9 of 11 experiments), effects of tDCS on inhibitory control was investigated [6, 68–74] and in 5 of 10 studies (or 6 of 11 experiments), tDCS effects on WM were explored [6, 69, 74–76]. In what follows, we describe the effect size obtained from studies to examine the efficacy of tDCS in ADHD major neuropsychological symptoms (Please see Fig 2). Brief details of each study are also summarized in Tables 1 and 2.

### Risk of bias

The authors’ judgment on the risk of bias is displayed in Fig 1B. Overall, the risk of bias was low. However, uncertainty about the risk does exist, at least to a certain extent, for selection bias, performance bias, and detection bias. Especially, for determination of the latter, information was missing in the majority of studies. A selective reporting bias was observed for the study of Munz et al. [72] since accuracy data for the experimental conditions are not reported in the manuscript, nor in any appendices. In all studies, blinding of participants was reported, but blinding of experimenters was not reported in two studies [70, 73]. We need to note that true blinding procedure of participants requires debriefing at the end of treatment about the active or sham mode of stimulation confirmed by statistical analysis. This was reported or confirmed only in three included studies implicating that blinding of participants should be interpreted with caution. All studies used randomization to allocate participants to different experimental conditions.

### Effects of tDCS on inhibitory control in ADHD patients

A significant cumulative effect size (Ē) of 0.197 (Z = 2.76, p = 0.006) was observed for a general tDCS effect on inhibitory control, taking polarity not into account Kolmogorov-Smirnov’s test of normality showed that the distribution of the effect sizes was not significantly different from a normal distribution (lower bound *p* = 0.20) and total heterogeneity of the effect sizes was not significant (Qtotal = 45.21, p = 0.463). The fail-safe number indicated that 221 unpublished null-findings would be required to render the effect non-significant. Exploration of montage showed that only dlPFC stimulation (l-dlPFC and bilateral) (Ē = 0.243, Z = 2.92, p = 0.004), but not rIFG stimulation (Ē = 0.005, Z = 0.04, p = 0.971) yielded a significant increase of accuracy rates in inhibitory control task performance.

Subsequently, polarity-dependent effects were investigated. Studies using anodal tDCS showed a significant Ē of 0.232 (Z = 2.72, p = 0.007), with a fail-safe number of 171 showing that anodal tDCS significantly improved inhibitory control. This sample was distributed normally (lower bound *p* = .20) and showed no significant heterogeneity (Qtotal = 33.05, p = 0.465). As for the stimulation polarity-independent analysis, this effect was driven by studies using a left and bilateral dlPFC montage (Ē = 0.255, Z = 2.66, p = 0.008), whereas the rIFG montage did not yield a significant effect (Ē = 0.084, Z = 0.33, p = 0.745). In contrast to anodal tDCS, cathodal tDCS did not show a significant overall effect (Ē = 0.089, Z = 0.63, p = 0.530).

Finally, an analysis was performed separating outcomes measures that focused on accuracy or amount of errors compared to the speed of response. The results showed that tDCS significantly increased accurate responses in inhibitory control tasks (Ē = 0.250, Z = 2.36, p = 0.019). No significant cumulative effect was found for speed (Ē = 0.093, Z = 1.09, p = 0.278). For this last analysis, a deviation from normality was observed (p = 0.031). Results are summarized in Table 3.

**Table 3:** Meta-analysis results for the effects of tDCS on inhibitory control in ADHD patients.

Cumulative effect size							Normality		Heterogeneity	
Analysis	N	Ē	95% CI	Z	p-v0alue	Fail-safe number	KS test	p-value	Qtotal	p-value
Polarity-independent										
All studies	46	**0.197**	**0.057–0.336**	**2.758**	**0.0058**	221	0.088	LB 0.200	45.21	0.463
dlPFC only	38	**0.243**	**0.080–0.406**	**2.922**	**0.0035**	97	0.077	LB 0.200	36.87	0.475
rIFG only	8	0.005	-0.261–0.271	0.037	0.9705	0	0.195	LB 0.200	6.411	0.493
Polarity-dependent										
Anodal tDCS	34	**0.232**	**0.065–0.400**	**2.723**	**0.0065**	171	0.095	LB 0.200	33.05	0.465
dlPFC only	30	**0.255**	**0.067–0.443**	**2.658**	**0.0079**	56	0.085	LB 0.200	28.98	0.466
rIFG only	4	0.084	-0.422–0.589	0.325	0.7452	0	^1^		2.25	0.523
Cathodal tDCS	12	0.089	-0.189–0.367	0.628	0.5300	0	0.133	LB 0.200	11.27	0.421
dlPFC only	8	0.194	-0.212–0.600	0.937	0.3488	0	0.114	LB 0.200	7.06	0.423
rIFG only	4	-0.075	-0.635–0.486	-0.263	0.7926	0	^1^		3.01	0.390
Speed vs Accuracy										
Accuracy	27	**0.250**	**0.042–0.459**	**2.356**	**0.0185**	36	0.096	LB 0.200	26.30	0.447
Speed	19	0.093	-0.075–0.261	1.085	0.2779	0	**0.208**	**0.031**	18.08	0.451

tDCS = Transcranial Direct Current Stimulation; dlPFC = dorsolateral prefrontal cortex; LB = lower bound; rIFG = right inferior frontal gyrus; Ē = cumulative effect size; CI = Confidence interval; KS = Kolmogorov-Smirnov’s test of normality; Qtotal = total heterogeneity represented by Cohen’s Q; Significant results are highlighted in bold. dlPFC refers to either left dlPFC or bilateral dlPFC (for detailed information refer to Tables 1 and 2 under tDCS montage column). 1KS test could not be performed because of too small sample size

### Effects of tDCS on working memory in ADHD patients

No significant cumulative effect was observed for tDCS on working memory, without taking polarity into account (Ē = 0.160, Z = 0.81, p = 0.418). Also, no effect of tDCS was observed when only studies with an anodal montage were included (Ē = 0.116, Z = 0.53, p = 0.600). However, when separating outcomes for accuracy and speed, a significant effect of tDCS on speed was observed. TDCS led to a faster response time (Ē = 0.681, Z = 2.43, p = 0.015), with a fail-safe number of 17. The sample was normally distributed (lower bound *p* = .20) and no significant heterogeneity was seen (Qtotal = 5.88, p = 0.437). These results should be interpreted with caution, given the low sample size (N = 7). Moreover, results showed that tDCS did have no significant effect on accuracy of working memory task performance (Ē = -0.187, Z = -0.76, p = 0.446). Results are shown in Table 4.

**Table 4:** Meta-analysis results for the effects of tDCS on working memory in ADHD patients.

Cumulative effect size							Normality		Heterogeneity	
Analysis	N	Ē	95% CI	Z	p-value	Fail-safe number	KS test	p-value	Qtotal	p-value
Polarity independent										
All studies	18	0.160	-0.227–0.547	0.811	0.4176	0	0.111	> 0.200	17.36	0.430
Polarity-dependent										
Anodal tDCS	16	0.116	-0.317–0.549	0.525	0.5996	0	0.107	> 0.200	15.46	0.419
Speed vs Accuracy										
Accuracy	11	-0.187	-0.668–0.295	-0.762	0.4461	0	0.163	> 0.200	10.09	0.433
Speed	7	**0.681**	**0.131–1.231**	**2.427**	**0.0152**	17	0.130	> 0.200	5.88	0.437

tDCS = Transcranial Direct Current Stimulation; Ē = cumulative effect size; CI = Confidence interval; KS = Kolmogorov-Smirnov’s test of normality; Qtotal = total heterogeneity represented by Cohen’s Q; Significant results are highlighted in bold

## Discussion

ADHD is a major neurodevelopmental disorder with remarkable heterogeneity in symptoms, etiologies, and treatment response. Recent studies have emphasized on executive dysfunctions and brain functional abnormalities underlying ADHD pathophysiology [14, 20, 54, 57] suggesting that modulation of cortical activity in the involved brain regions via tDCS might ameliorate neuropsychological symptoms [77]. Exploring the impact of tDCS especially on ADHD neuropsychological deficits has significantly increased in the last few years. However, no meta-analysis about tDCS efficacy in ADHD is available. We conducted a meta-analysis of randomized, placebo-or-baseline-controlled trials of tDCS application in ADHD, which is the first meta-analysis for ADHD patients.

Overall, results of this meta-analysis show that tDCS improved inhibitory control and WM in ADHD. Further sub-analyses yielded the following findings: (1) tDCS has an overall significant cumulative effect on inhibitory control in ADHD with a small effect size, (2) when the targeted brain region is taken into account, only tDCS over the dlPFC had a significant effect on inhibitory control (small-to-medium effect size), but not tDCS over the rIFG, (3) when stimulation polarity was taken into account, only anodal, but not cathodal tDCS had a significant effect on inhibitory control, (4) when both polarity and targeted region are taken into account, only anodal tDCS of the dlPFC had a significant effect on inhibitory control with a small-to-medium effect size, (5) and when analyzing inhibitory control outcomes separately, tDCS had a significant cumulative effect on accuracy, but not speed (i.e., reaction time). With regard to WM (6), tDCS had an overall significant effect only on performance speed but not on accuracy. In what follows we discuss tDCS effects on these two major target deficits, the stimulation parameters that could yield to larger effects, and finally clinical and methodological implications for future studies.

### tDCS effects on inhibitory control in ADHD

The significant overall effect of tDCS on inhibitory control in ADHD is in line with a previous meta-analysis about the effects of this technique on a variety of cognitive functions (e.g., WM, executive functions, language) [78–80] and neuropsychiatric populations [80–82]. Moreover, the small effects size of the overall effect of tDCS found in our meta-analysis is consistent with small-to-medium reported effect sizes in previous meta-analyses about the effectiveness of tDCS [78, 82–84]. However, the effects of tDCS on inhibitory control were associated with larger effect size when target area (i.e., electrode positioning) and stimulation polarity (anodal vs cathodal) were taken into account.

Anodal dlPFC tDCS had the largest effect size (small-to-medium effect) on inhibitory control in ADHD populations, whereas anodal rIFG tDCS had no significant effect. For stimulation over the dlPFC, both left and right dlPFC were targeted in different studies but the left dlPFC was almost always stimulated with the target electrode. First of all, this is in line with previous findings about significant involvement of the dlPFC in ADHD symptoms, especially executive dysfunctions [16, 18]. Inhibitory control is a major component of dlPFC-supported executive functioning [85, 86] which is impaired in ADHD. The dlPFC is hypoactive in ADHD, which is associated with insufficient suppression of dlPFC signaling not only in the resting state, but also during the performance of cognitive tasks [20, 87]. Recent meta-analyses of neuroimaging studies about functional abnormalities in ADHD populations showed a bilateral hypoactivity of the dlPFC [88] and reduced left medial frontal cortex activation [89] in ADHD populations during inhibitory control, WM and attention task performance. This hypoactivity of the dlPFC is assumed to underlie the attentional deficit, impaired inhibitory control and executive dysfunctions in ADHD and therefore, the pathophysiological rationale for the therapeutic application of tDCS is to increase dlPFC activation with anodal stimulation. It further supports the “cognitive dysfunction or inhibition-based model” of ADHD which suggests that inhibition-based executive deficits are a core deficit in ADHD [56, 90].

Secondly, it highlights the importance of stimulation site for the tDCS effects on inhibitory control. Our findings showed that dlPFC tDCS and not rIFG tDCS is effective. Moreover, the target area of the reference electrode impacts on tDCS effects on inhibitory control. TDCS effects on inhibitory control were more effective when two specific regions were stimulated by the target and reference electrodes. The left dlPFC-right OFC montage was the most effective montage and all of the four experiments which used this protocol reported improved performance. In contrast, the left dlPFC-right dlPFC tDCS montage, which was used in two studies, did not improve any inhibitory control outcome measure. In two studies that targeted only one prefrontal region (i.e., left dlPFC-Cz tDCS, left/right dlPFC- mastoid tDCS), inhibitory control was also improved (see Table 5). Thirdly and in addition to the stimulation site, the results of this meta-analysis demonstrate the importance of stimulation polarity in the tDCS effects on inhibitory processes in ADHD. Anodal tDCS, especially over dlPFC yielded the largest effect size for improving inhibitory control in ADHD. Even the two studies that reported significant effects of cathodal tDCS on response inhibition, argued that cathodal tDCS over the left dlPFC was probably effective in improving inhibitory control in ADHD by increasing activity of the right dlPFC through the inhibitory link between contralateral dlPFC regions via the transcallosal connections. That being said, further systematic investigations are still required to more robustly specify the contribution of potential stimulation sites and polarity on response inhibition in ADHD populations.

**Table 5:** Characteristics of the stimulation site and polarity in the studies investigated tDCS effects on inhibitory control and working memory.

Inhibitory control				
#	Authors	Stimulation target (site)	polarity	Performance improvement
1	Breitling et al (2016)	right IFG—left mastoid	Anodal	No
2	Soltaninejad et al (2015)	Left dlPFC—right supraorbital area (OFC)	Anodal	No
		Left dlPFC—right supraorbital area (OFC)	Cathodal	Yes
3	Allenby et al (2018)	Left dlPFC—right supraorbital area (OFC)	Anodal	Yes
4	Bandeira et al (2016)	Left dlPFC—right supraorbital area (OFC)	Anodal	Yes
5	Nejati et al (2017) Exp 2	Left dlPFC—right supraorbital area (OFC)	Anodal	No
		Left dlPFC—right supraorbital area (OFC)	Cathodal	Yes
6	Nejati et al (2017) Exp 1	Left dlPFC—right dlPFC	Anodal	No
7	Cosmo et al (2015)	Left dlPFC—right dlPFC	Anodal	No
8	Sotnikova et al (2017)	Left dlPFC—vertex	Anodal	Yes
9	Munz et al (2015)	Left dlPFC–mastoid	Anodal	Yes (only RT)
		right dlPFC—mastoid	Anodal	Yes (only RT)
**Working memory**				
1	Soff et al. (2017)	Left dlPFC—vertex	Anodal	Yes (both accuracy and RT)
2	Sotnikova et al (2017)	Left dlPFC—vertex	Anodal	Yes (both accuracy and RT)
3	Nejati et al (2017) Exp 1	Left dlPFC—right dlPFC	Anodal	Yes (only RT)
4	Nejati et al (2017) Exp 2	Left dlPFC—right supraorbital area (OFC)	Anodal	Yes (both accuracy and RT)
		Left dlPFC—right supraorbital area (OFC)	Cathodal	No
5	Bandeira et al (2016)	Left dlPFC—right supraorbital area (OFC)	Anodal	No
6	Prehn-Kristensen et al (2014)	Left dlPFC / right dlPFC–mastoid	Anodal	Yes

tDCS = transcranial direct current stimulation; dlPFC = dorsolateral prefrontal cortex; IFG = inferior frontal gyrus; OFC = orbitofrontal cortex.

Additionally, the findings suggest that heterogeneity of ADHD subtypes and brain abnormalities should not be neglected in interpreting the tDCS effects. This heterogeneity could explain why rIFG tDCS was not as effective as dlPFC tDCS in the present analysis. For example, both, rIFG and left/right dlPFC abnormalities are shown to be associated with inhibitory control deficits in ADHD [13, 18, 53]. However, a differential involvement of the dorsal/ventral divisions within the lateral PFC is suggested in ADHD pathophysiology. Dorsal regions (i.e., dlPFC) are suggested to support inhibitory control of cognitive processes, while more ventral regions (e.g., rIFG) support motor domains of inhibitory control [18, 91]. This shows that ADHD subtypes (i.e., inattention type vs hyperactive type) are important for variations in tDCS effects and implicates that the rIFG tDCS might be well-suited for those groups of patients with more hyperactive than inattentive symptoms. Moreover, dlPFC has a global contribution to executive functioning and response inhibition regardless of modality [42]. This global contribution of the dlPFC in all aspects of response inhibition might explain the significant implication of the dlPFC, but not rIFG in ADHD inhibitory processes. Furthermore, ADHD is a disorder with disturbances in large-scale inter-related brain networks [23, 24]. The dlPFC is a crucial part of the prefrontal network and compared to other prefrontal regions, has many connections with other cortical/subcortical areas [92, 93]. It is possible that dlPFC stimulation effectively hits the entire prefrontal network including the dlPFC-rIFG sub-network, whereas, rIFG stimulation has more restricted effects. Such a global contribution of the dlPFC to other prefrontal region functions has been shown for various executive functions [42] which might be true for ADHD too.

### tDCS and WM performance in ADHD

Our meta-analysis showed that tDCS does not have an overall significant effect on WM performance accuracy in ADHD, although it led to significantly faster response time. To be more specific, it was shown that anodal left dlPFC tDCS, but not cathodal stimulation improved response time during WM performance in ADHD. This finding should be discussed considering physiological processes underlying inhibitory control and WM, task characteristics, and results of previous meta-analyses about tDCS effects on WM. First of all, this result shows a dissociation of tDCS effects on inhibitory control and WM. Such a dissociation has also been shown for neuropsychological deficits in ADHD [53]. Inhibitory control is an executive control function [42] and requires executive attention to inhibit a prepotent response and monitoring, whereas memory tasks can deploy attentional resources [53]. Different effects of tDCS on inhibitory control and WM thus could be attributed to different cognitive processes underlying WM and inhibitory control as well as target area-specific effects of tDCS, which need to be studied in future systematically. Characteristics of the respective WM tasks can also affect the results. For example, performance on the n-back task can still be faster while accuracy cannot due to ceiling effects in case of near-optimal accuracy already without intervention.

Another factor that could be a source of variance of the tDCS effects on WM in the included studies is the difference of stimulation sites which is an important feature of tDCS efficacy. The stimulation montages of the studies on WM included anodal left dlPFC-right OFC tDCS (two experiments), left dlPFC-right dlPFC tDCS (two experiments), and left dlPFC-vertex tDCS (two experiments). Different montages used in these studies can explain the observed effects and may explain why some studies demonstrated no effects on WM accuracy. In four of the experiments, the reference electrode was positioned over regions that are involved in cognitive performance (i.e., right OFC and right dlPFC). In this case, it might be speculated that the cathodal return electrode compromises performance, and thus partially antagonizes anodal tDCS effects over the target region. The other two experiments used unilateral protocols that targeted the mere region of interest without positioning the cathodal return electrode over a potentially involved region and interestingly, in these studies both WM accuracy and RT improved, and prolonged effects outlasting the stimulation were also reported. Therefore, an electrode positioning which avoids such antagonistic effects might be advantageous.

Recent meta-analyses and review articles about the effects of tDCS on cognitive functioning showed mixed results regarding memory tasks. For example, a recent systematic review of 188 trials reported significant effects of anodal tDCS on RT only for executive functioning tasks, but not memory tasks in both, healthy and neuropsychiatric populations [80]. Similar to our finding, another meta-analysis of 12 tDCS WM trails found that tDCS significantly improved RT, but not accuracy [78]. A more recent meta-analysis about the effects of tDCS on WM (measured with n-back and digit span tasks) found a significant effect of anodal tDCS on accuracy and RT for offline, but not online tasks in healthy subjects; in neuropsychiatric populations, tDCS had a significant effect on WM accuracy (both offline and online) but not RT [84]. Finally, a recent meta-analysis of 61 studies investigated the effect of tDCS on cognitive tasks in relation to task and stimulation parameters. They found that in contrast to offline-task performance, online-task performance resulted in increased accuracy in clinical populations [94]. Interestingly, in 5 out of 6 studies included in our meta-analysis for WM, offline task performance was measured in ADHD individuals. Thus, the non-significant effect of tDCS on WM accuracy we found could be due to offline task performance in the majority of studies, which might be less efficient. Nevertheless, the results of the effect of tDCS on WM in ADHD should be treated with caution due to the limited number of qualified studies that did not allow us to disentangle polarity- and area-dependent effect of tDCS.

### Clinical and methodological implications

This meta-analysis found that modulation of the activity of brain areas involved in the pathophysiology of ADHD with tDCS has a significant effect on response inhibition and WM, two major executive dysfunctions in ADHD [14, 53, 54]. This suggests that tDCS over the dlPFC might be suited as a potential therapeutic approach in ADHD, similar to other neuropsychiatric syndromes which are responsive to this method [45]. Increasing evidence from clinical and cognitive neuroscience describes ADHD as a disorder with functional/structural abnormalities, especially of the frontostriatal circuitry and PFC. Accordingly, modulation of the involved brain areas with non-invasive brain stimulation is a promising approach for improving neuropsychological symptoms in ADHD patients. The efficacy of this method might, however, depend on ADHD subtypes/symptoms and stimulation parameters, which need to be investigated systematically in future studies.

The findings of this meta-analysis have some important clinical and methodological implications to be considered for future tDCS studies in ADHD. The results of the included studies showed beneficial effects of tDCS in ADHD, and at the same time indicate that effects of tDCS on ADHD deficits (namely inhibitory control and WM) are strongly dependent on 1) individual and inter-individual factors (e.g., type of symptoms/deficits) and 2) also stimulation parameters (i.e., site or cortical target, polarity, intensity, duration, and repetition rate). For example, heterogeneous ADHD subtypes in the analyzed studies (i.e., inattentive or hyperactive) might partially explain the non-significant effect of anodal rIFG tDCS on response inhibition, since dlPFC activity contributes to response inhibition in all ADHD subtypes (especially individuals with inattentive symptoms), while the rIFG is assumed to be specifically involved in ADHD individuals with more hyperactivity subtypes. Therefore, the inter-individual variability is an important factor which generally affects the efficacy of tDCS [95] and might be specifically relevant for the treatment of ADHD patients, that vary in subtypes. Furthermore, findings of those studies that investigated tDCS effects on other ADHD deficits showed that for those EF domains that involve motivational and emotional processing, tDCS over both, prefrontal and frontopolar areas is more effective compared to dlPFC-only tDCS. For example, the Wisconsin Card Sorting Test (WCST) is a well-documented measure of EF and primarily measures cognitive flexibility. This task involves aspects of both, hot (e.g., task switching, disinhibition, WM) and cold EFs (i.e., executive control, inhibition) and results showed that only when both, dlPFC and OFC were stimulated, cognitive flexibility improved. Therefore, the common stimulation protocols in ADHD that primarily target dorsolateral frontostriatal networks may not be ideal for all executive dysfunction in ADHD, especially hot executive dysfunctions. This highlights the necessity of symptom-driven protocols implicating that for each specific impairment (e.g., response inhibition, WM, selective attention, interference control, cognitive flexibility, etc.) a specific stimulation montage that targets the most relevant cortical region would be most appropriate.

Another important implication regarding the clinical efficacy of tDCS in ADHD concerns stimulation parameters (i.e., intensity, duration, repetition rate, polarity, and site). The stimulation intensities applied in the included studies ranged from 1 mA (seven experiments) to 2 mA (two experiments) and two experiments applied tDCS with 1.5 mA intensity. Findings from other clinical populations showed that higher intensities of stimulation can result in more prominent symptom improvement, for example in tinnitus [96], or cognitive impairment in Parkinson’s disease [97]. This is in further accordance with physiological findings of tDCS about larger tDCS after-effects in motor cortex plasticity as a result of tDCS with higher intensities [98]. This was supported by the findings from two studies conducted on adult ADHD. The study with 2 mA intensity reported significant improvement in inhibitory control performance [68] while the study with 1 mA intensity reported no significant effects on Go/No-Go task performance [71] despite the fact both studies targeted left dlPFC with anodal tDCS. Stimulation intensity has, therefore, an impact on the effectiveness of tDCS, which is not necessarily linear [99] and should be considered in clinical applications.

With regard to stimulation duration, the length of stimulation duration varied from 8 min to 30 min and the longest duration of stimulation reported was five consecutive days of 30-min stimulation (Tables 1 & 2). The effects of different stimulation durations were not addressed systematically in ADHD studies so far. Longer stimulation duration, similar to stimulation intensity, could be an important factor to improve the efficacy of tDCS effects, including clinical effects. Prolongation of stimulation duration for increasing efficacy of the intervention works similarly well as enhancing stimulation intensity. One advantage of enhancing stimulation duration compared to intensity might be that this does not increase the probability of side effects like itching and tingling [33, 100], which might be specifically relevant for studies in children. Findings from other neuropsychiatric disorders show that repeated sessions of tDCS are more effective in reducing symptoms [33]. All studies included in this meta-analysis except one [69] examined effects of tDCS after one single session. Higher clinical efficacy might be achieved with a longer duration of stimulation, and repetitive interventions, as shown for example in non-invasive brain stimulation studies in depression [101–103].

Stimulation polarity is another important parameter that determines the efficacy of tDCS. The physiological mechanism of tDCS effects, depending on the polarity, is the induction of LTP- and LTD-like plasticity [36, 104]. Anodal tDCS induces LTP-like plasticity, based on subthreshold depolarization effects on membrane potentials, and respective enhancement of spontaneous neuronal activity, while cathodal tDCS has antagonistic effects [105]. In accordance with the pathophysiological foundation of ADHD that includes underactivation of the lateral and inferior PFC, anodal tDCS-generated excitability enhancement is conceptually a more promising approach in ADHD. The findings of this meta-analysis are in accordance with this assumption and showed that anodal, but not cathodal tDCS improved inhibitory control and WM in ADHD which could be attributed to the stimulation-induced compensation for regional cortical hypoactivity as well as alteration of functional cortical network connectivity following anodal tDCS that improves cognitive performance [106]. Nevertheless, this does not mean that cathodal tDCS is not of clinical interest in ADHD. Functional abnormalities in ADHD are not limited to hyperactivation of specific regions but also hyper-sensitiveness of other regions, especially those involved in motivational and emotional processing [11]. Moreover, findings from cathodal stimulation of the motor cortex showed that motor cortex excitability alterations induced by tDCS are intensity-dependent and nonlinear [99] which is not well studied for non-motor functions. It might be that cathodal tDCS over regions pathologically hyperactive in the disease might also be beneficial, which has not been systematically explored so far.

The cortical target area or stimulation site, another important parameter, contributes to the clinical efficacy of tDCS in ADHD. The contribution of this parameter was specifically clear in the studies which investigated tDCS effects on ADHD WM deficits. Only when the potentially involved cortical region (left dlPFC in WM) was stimulated with the target electrode, both WM accuracy and RT significantly improved and the effects were long-lasting. In these cases, the reference electrode was placed over a non-contributing area (i.e., vertex). In contrast, when the reference electrode targeted homologous regions of the contralateral hemisphere (i.e., right dlPFC or right supraorbital) the effects were not present or reduced to RT improvement only. A comparable pattern but in a different way was observed for tDCS effects on inhibitory control. Here, tDCS effects were more effective when two specific potentially involved regions were stimulated by the target and reference electrodes. The left dlPFC-right OFC montage was the most effective montage and all of the four experiments which used this protocol reported improved performance. In contrast, the left dlPFC-right dlPFC tDCS montage, which was used in two studies, did not improve any inhibitory control outcome measure. This demonstrates that the target area or areas significantly contribute to the tDCS effectiveness in ADHD.

To conclude, results from this meta-analysis support the cognitive benefits of tDCS in ADHD. However, for these cognitive benefits to be clinically useful, effects need to be sustained for longer durations. All of the studies included in this meta-analysis applied tDCS in an acute and short-term mode with no follow-up examination which does not allow to infer long-term efficacy of tDCS in improving ADHD neuropsychological deficits. Similarly, measuring inhibitory control and WM was done either during stimulation or right/shortly after intervention which hinders any robust conclusions about long-term effects of tDCS. One specific important note here is the dissociation between neuropsychological deficits and clinical symptoms of ADHD, which means improvement in inhibitory control and WM after or during tDCS does not say too much about the improvement of clinical symptoms. Moreover, applying statistically-proven blinding procedures, which was not reported in most of the included studies in this meta-analysis, is important for making firm conclusions about the clinical efficacy of tDCS. Therefore, the results of this meta-analysis should be interpreted with caution, especially when it comes to the clinical efficacy of tDCS in ADHD. Nevertheless, we need to note that the studies included in this meta-analysis are actually the first tDCS studies on ADHD that primarily aimed to investigate whether tDCS has any beneficial effects on ADHD symptoms or not. Despite short-term tDCS-induced cognitive benefits in ADHD, the clinical efficacy of this technique needs further investigation in follow-up designs and in comparison to other interventions. Moreover, it requires stimulation protocols optimization using the information about the individual neural activity associated with deficits and task execution (individual level) and general stimulation parameters [49].

### Safety of tDCS

There are unique issues concerning the safety, applicability, and ethics of tDCS application in pediatric populations which is mainly due to limited available data from children compared to the adult population [52]. However, the general safety of tDCS with standard protocols has been proven by a large body of evidence in recent years and a recent review concluded that application of conventional tDCS in human trials has not yet produced any reports of a Serious Adverse Effect or irreversible injury [107]. In pediatric populations, no severe adverse events have been reported, and even in children with epilepsy, seizures do not seem to worsen with tDCS [52]. The most frequently reported side-effects within studies included in that systematic review were headache, itchiness, and redness at the site of the stimulation. Similarly, no significant side-effects were reported in the studies included in our meta-analysis. This is important due to some concerns about the application of tDCS in children. One specific concern is about current intensity due to children’s thinner skulls and the smaller distance between scalp and brain. Computational models of current flow within the brain suggest that about 50% of the current strength applied in adults result in respective effects in children [52] implicating that 0.5 mA applied in children results in similar physiological effects as 1 mA in adults [108]. From the studies included in this meta-analysis, eight studies applied a current intensity of 1 mA or lower, two studies applied 1.5 mA and one study applied 2 mA, and no significant adverse effect was reported (See Tables 1 & 2). However, the caveat still stands that studies included in this meta-analysis were not designed as safety studies and mostly included single session tDCS interventions. Therefore, systematic safety monitoring, especially for the clinical application of tDCS, is recommended. Moreover, it is important to keep in mind that the developing brain has “sensitive” or “critical” periods where the effects of interventions affecting the brain could be stronger than usual. This suggests that the risk to induce maladaptive neural plasticity due to tDCS might be high which necessitates the priority of dose-finding studies and longitudinal monitoring of tDCS-induced neuroplasticity in pediatric ADHD population [49].

### Limitations and future directions

Despite promising results and novelty, this meta-analysis has some limitations to be considered. First, despite that our meta-analysis found a significant effect of tDCS on neuropsychological symptoms (namely inhibitory control and WM) in ADHD populations, the efficacy of this method on other executive dysfunctions, and clinical symptoms cannot be directly derived from these results. Inhibitory control and WM were chosen because they are two common deficits examined in most ADHD-tDCS studies which allowed us to run a meta-analytic study. Secondly, although we showed that anodal tDCS over the dlPFC has significant effects on IC in ADHD, no effects were seen for cathodal tDCS and rIFG tDCS. However, given the small number of studies investigating cathodal/rIFG tDCS, these results have to be interpreted with caution. With increasing interest in the application of tDCS in ADHD, future research might be able to disentangle subtle protocol differences for ADHD subtypes. Thirdly, adaptation of stimulation protocols based on symptom subtypes, ADHD subgroups, specific cognitive deficits, and neuroanatomical differences is lacking in currently available studies and therefore, it is difficult to rate the potential of tDCS in ADHD.

Future tDCS studies should systematically investigate the following lines of research, to reveal the real clinical potential of tDCS for ADHD treatment. First, preliminary data suggest beneficial effects of tDCS on other cognitive dysfunctions in ADHD including cognitive flexibility, problem-solving, selective attention, and hyperactivity symptoms involved in ADHS [6, 70, 109]. Second, based on the findings of this meta-analysis, future tDCS studies on ADHD populations, with a statistically-proven blinding procedure (especially double-blinded trials), are recommended to target the stimulation area based on the symptoms subtypes. The above-mentioned stimulation parameters are other important factors that might affect the efficacy of the intervention and should be explored systematically. Lastly, future studies, especially those with clinical implications, require examining the long-term efficacy of tDCS in ADHD. With the expected increasing number of tDCS studies in ADHD populations, future meta-analytic studies might be able to deliver a more realistic picture about the effectiveness of tDCS in different subtypes of ADHD. It is lastly of note that other modalities of electrical stimulation such as transcranial alternating current stimulation (tACS), which has been shown to have neuroplastic effects beyond its impact on brain oscillations [110], or transcranial random noise stimulation (tRNS) can be a potentially interesting avenue for future research in ADHD, which is associated with abnormal brain oscillations [24].

### Conclusion

The findings of this meta-analysis of tDCS interventions in ADHD suggest an improvement of neuropsychological deficits (i.e., inhibitory control and WM) by tDCS. Stimulation polarity and target area are relevant for the efficacy of tDCS in ADHD. Anodal dlPFC tDCS had a significantly superior effect on inhibitory control compared to cathodal/sham stimulation and anodal rIFG tDCS. TDCS significantly increased response accuracy of inhibitory control performance and decreased response time in WM tasks. Although our findings suggest improving effects of tDCS in ADHD neuropsychological deficits, the clinical utility of tDCS cannot be firmly rated with the currently available findings. Application of this method as a therapeutic intervention will require optimizing stimulation protocols based on general stimulation parameters and individual and inter-individual factors for improvement of clinical efficacy, exploration of clinical symptoms in addition to surrogate parameters, and achievement of sustained clinical benefits by tDCS over longer durations of time. Thus, future research is needed to more thoroughly explore and refine optimal stimulation parameters required for tDCS-based cognitive improvement and implementing robust experimental designs in different ADHD subtypes. Broadly speaking, the potential for tDCS as a non-invasive brain stimulation technique to safely improve neuroplasticity and treat neurological and neurodevelopmental disorders is encouraging. Future studies utilizing tDCS will further increase our understanding of neural networks and how to treat their pathological states in ADHD and other neurodevelopmental disorders including autism and learning disabilities.

## Supporting information

S1 Table The Preferred Reporting Items for Systematic reviews and Meta-Analyses (PRISMA) 2009 checklist. (DOC)
