# Correction: Transcranial direct current stimulation in attention-deficit hyperactivity disorder: A meta-analysis of neuropsychological deficits

**DOI:** 10.1371/journal.pone.0221613

**Published:** 2019-08-20

**Authors:** Mohammad Ali Salehinejad, Miles Wischnewski, Vahid Nejati, Carmelo M. Vicario, Michael A. Nitsche

The values of three out of the ten studies (bolded in the Tables) included in this meta-analysis were entered incorrectly due to copy-pasting errors. As a result, there are errors in Tables 1–4, Fig 2, and the Abstract, Results, and Discussion sections. The authors confirm that these modifications do not alter the conclusions of the study.

In Table 1, there are errors in results under the column titled “Hedges’ g” for the studies “Cosmo et al (2015)”, “Nejati et al (2017) experiment 1”, “Nejati et al (2017) experiment 2”, and “Sotnikova et al (2017)”.

In Table 2, there are errors in results under the column titled “Hedges’ g” for the studies “Nejati et al (2017) experiment 1” and “Nejati et al (2017) experiment 2”.

In Tables 3 and 4, there are errors in the results reported in the “Analysis” rows under the column subheadings of “Ē”, “Z”, “p-value”, “Fail-safe number”, “KS test”, “Qtotal”, and “p-value”.

Please see the correct Tables 1–4 here.

**Table 1:** Characteristic of studies included in meta-analysis for the effecs of tDCS on inhibitory control.

#	Authors	N	Mean age	tDCS montage (target/reference)	Intensity	Duration	Polarity	On-/off-line	Control	Task	Outcome	Hedges’ g
1	Allenby et al (2018)	37	37.17 (range 18–56)	F3/Fp2 (25 cm^2^ both)	2 mA	3 days x 20 min	Anodal	Offline	Baseline + sham	CPT	False positive errors	0.42
											True positive errors	-0.06
											Response time	-0.11
				F3/Fp2 (25 cm^2^ both)	2 mA	3 days x 20 min	Anodal	Offline	Baseline + sham	SST	Reaction time	-0.18
2	Bandeira et al (2016)	9	11.1 ± 2.8	F3/Fp2 (35 cm^2^ both)	2 mA	5 days x 30 min	Anodal	Offline	Baseline	NEPSY II	Total errors	0.12
											Completion time	0.54
3	Breitling et al (2016)	21	14.33 (range 13–17)	F8/mastoid (35 cm^2^ both)	1 mA	20 min	Anodal	Online	Sham	Flanker task	Omission errors	-0.11
											Comission errors	0.46
											Reaction time	-0.14
											Reaction time variability	0.13
				F8/mastoid (35 cm^2^ both)	1 mA	20 min	Cathodal	Online	Sham	Flanker task	Omission errors	-0.60
											Comission errors	0.17
											Reaction time	0.13
											Reaction time variability	-0.02
4	Cosmo et al (2015)	30	31.8 ± 11.6	F3/F4 (35 cm^2^ both)	1 mA	20 min	Anodal	Offline	Sham	Go/No-go task (letters)	Correct responses	0.06
											Omission errors	-0.08
											Comission errors	0.26
										Go/No-go task (fruits)	Correct responses	-0.12
											Omission errors	-0.05
											Comission errors	-0.15
5	Munz et al (2015)	14	12.3 ± 1.4	F3+F4/both mastoids (0.5 cm^2^ all)	0–0.25 mA (oscillatory)	5 x 5 min	Anodal	Offline	Sham	Go/No-go task	Reaction time	0.88
											Reaction time variability	0.83
6	Nejati et al (2017) experiment 1	15	10 ± 2.2	F3/F4 (25 cm^2^ both)	1 mA	15 min	Anodal	Offline	Sham	Go/No-go task	Go accuracy	0.13
											No-go accuracy	0.08
											Reaction time	0.24
										Stroop task	Accuracy	0.70
											Reaction time	1.09
7	Nejati et al (2017) experiment 2	10	9 ± 1.8	F3/Fp2 (25 cm^2^ both)	1 mA	15 min	Anodal	Offline	Sham	Go/No-go task	Go accuracy	0.41
											No-go accuracy	0.66
											Reaction time	-0.24
				F3/Fp2 (25 cm^2^ both)	1 mA	15 min	Cathodal	Offline	Sham	Go/No-go task	Go Accuracy	0.41
											No-go accuracy	1.21
											Reaction time	-0.68
8	Soltaninejad et al (2015)	20	Range 15–17	F3/Fp2 (35 cm^2^ both)	1.5 mA	8 min	Anodal	Offline	Sham	Go/No-go task	Go accuracy	-0.05
											No-go accuracy	0.03
											Reaction time	0.23
										Stroop task	Accuracy	0.57
											Reaction time	0.23
				F3/Fp2 (35 cm^2^ both)	1.5 mA	8 min	Cathodal	Offline	Sham	Go/No-go task	Go accuracy	-0.54
											No-go accuracy	0.73
											Reaction time	-0.02
										Stroop task	Accuracy	0.33
											Reaction time	0.11
											Reaction time	0.02
9	Sotnikova et al (2017)	13	14.33 ± 1.3	F3 (13 cm^2^)/ Cz (35 cm^2^)	1 mA	30 min	Anodal	Online	Sham	Go/No-go	accuracy (hits+correct rejections/total number of stimuli)	-0.68
											Reaction time	0.24
											Reaction time variability	-0.05

tDCS = transcranial direct current stimulation; F3 = left dlPFC; F4 = right dlPFC; F8 = inferior frontal gyrus; Fp1 = left supraorbital area; Fp2 = right supraorbital area; online = task performance during tDCS; offline = task performance after tDCS; CPT = Conners Continuous Performance Task; SST = Stop Signal Task (SST).

**Table 2:** Characteristic of studies included in the meta-analysis for the effects of tDCS on working memory.

#	Authors	N	Mean age	tDCS montage (target/reference)	Intensity	Duration	Polarity	On-/off-line	Control	Task	Outcome	Hedges’ g
1	Bandeira et al (2016)	9	11.1 ± 2.8	F3/Fp2 (35 cm^2^ both)	2 mA	5 days x 30 min	Anodal	Offline	Baseline	Digit span forward	Amount	-0.87
										Digit span backward	Amount	-0.40
										Corsi cube forward	Amount	-0.45
										Corsi cube backward	Amount	0.08
2	Nejati et al (2017) experiment 1	15	10 ± 2.2	F3/F4 (25 cm^2^ both)	1 mA	15 min	Anodal	Offline	Sham	1-back task	Accuracy	0.08
											Reaction time	1.39
3	Nejati et al (2017) experiment 2	10	9 ± 1.8	F3/Fp2 (25 cm^2^ both)	1 mA	15 min	Anodal	Offline	Sham	1-back task	Accuracy	1.15
											Reaction time	0.96
				F3/Fp2 (25 cm^2^ both)	1 mA	15 min	Cathodal	Offline	Sham	1-back task	Accuracy	0.54
											Reaction time	0.54
4	Prehn-Kristensen et al (2014)	12	12.1 (range 10–14)	F3+F4/both mastoids (0.5 cm^2^ all)	0–0.25 mA (oscillatory)	5 x 5 min	Anodal	Offline	Baseline + sham	Digit span	Amount	-0.61
5	Soff et al (2017)	15	14.2 ± 1.2	F3 (3.14 cm^2^)/Cz (12.5 cm^2^)	1 mA	5 days x 20 min	Anodal	Offline	Baseline + sham	QB (1-back) task	QB score (errors and reaction time)	0.50
6	Sotnikova et al (2017)	13	14.33 ± 1.3	F3 (13 cm^2^)/ Cz (35 cm^2^)	1 mA	30 min	Anodal	Online	Sham	1-back task	Accuracy	-0.99
											Reaction time	-0.05
											Reaction time variability	0.18
										2-back task	Accuracy	-1.14
											Reaction time	0.65
											Reaction time variability	1.06

tDCS = transcranial direct current stimulation; F3 = left dlPFC; F4 = right dlPFC; Fp2 = right supraorbital area; online = task performance during tDCS; offline = task performance after tDCS; QbTest = Quantified Behavior Test.

**Table 3:** Meta-analysis results for the effects of tDCS on inhibitory control in ADHD patients.

Cumulative effect size							Normality		Heterogeneity	
Analysis	N	Ē	95% CI	Z	p-value	Fail-safe number	KS test	p-value	Qtotal	p-value
Polarity-independent										
All studies	46	**0.117**	**0.008–0.252**	**2.104**	**0.0353**	79	0.105	LB 0.200	46.13	0.425
dlPFC only	38	**0.145**	**0.021–0.270**	**2.292**	**0.0219**	79	0.101	LB 0.200	38.16	0.417
rIFG only	8	0.005	-0.261–0.271	0.037	0.9705	0	0.195	LB 0.200	6.411	0.493
Polarity-dependent										
Anodal tDCS	34	**0.124**	**0.010–0.238**	**2.132**	**0.0330**	57	0.127	0.181	33.07	0.464
dlPFC only	30	**0.133**	**0.007–0.460**	**2.069**	**0.0385**	49	0.137	0.156	29.21	0.454
rIFG only	4	0.084	-0.422–0.589	0.325	0.7452	0	^1^		2.25	0.523
Cathodal tDCS	12	0.073	-0.231–0.378	0.471	0.6376	0	0.162	LB 0.200	11.90	0.371
dlPFC only	8	0.168	-0.297–0.634	0.708	0.4789	0	0.129	LB 0.200	7.65	0.364
rIFG only	4	-0.075	-0.635–0.486	-0.263	0.7926	0	^1^		3.01	0.390
Speed vs Accuracy										
Accuracy	27	0.113	-0.034–0.260	1.507	0.1319	0	0.125	LB 0.200	26.88	0.415
Speed	19	0.123	-0.054–0.300	1.390	0.1645	0	**0.224**	**0.013**	18.08	0.451

tDCS = Transcranial Direct Current Stimulation; dlPFC = dorsolateral prefrontal cortex; LB = lower bound; rIFG = right inferior frontal gyrus; Ē = cumulative effect size; CI = Confidence interval; KS = Kolmogorov-Smirnov’s test of normality; Qtotal = total heterogeneity represented by Cohen’s Q; Significant results are highlighted in bold. dlPFC refers to either left dlPFC or bilateral dlPFC (for detailed information refer to Tables 1 and 2 under tDCS montage column). 1KS test could not be performed because of too small sample size

**Table 4:** Meta-analysis results for the effects of tDCS on working memory in ADHD patients.

Cumulative effect size								Normality		Heterogeneity	
Analysis	N	Ē	95% CI	Z	p-value		Fail-safe number	KS test	p-value	Qtotal	p-value
Polarity independent											
All studies	18	0.150	-0.226–0.527	0.782		0.4342	0	0.121	LB 0.200	17.30	0.434
Polarity-dependent											
Anodal tDCS	16	0.103	-0.317–0.523	0.481		0.6307	0	0.109	LB 0.200	15.39	0.424
Speed vs Accuracy											
Accuracy	11	-0.192	-0.672–0.288	-0.784		0.4330	0	0.160	LB 0.200	10.12	0.430
Speed	7	**0.659**	**0.173–1.146**	**2.658**		**0.0079**	16	0.141	LB 0.200	5.88	0.437

tDCS = Transcranial Direct Current Stimulation; Ē = cumulative effect size; CI = Confidence interval; KS = Kolmogorov-Smirnov’s test of normality; Qtotal = total heterogeneity represented by Cohen’s Q; Significant results are highlighted in bold

Fig 2 has been corrected to reflect the updated results. Please see the corrected Fig 2 here.

**Fig 2:**
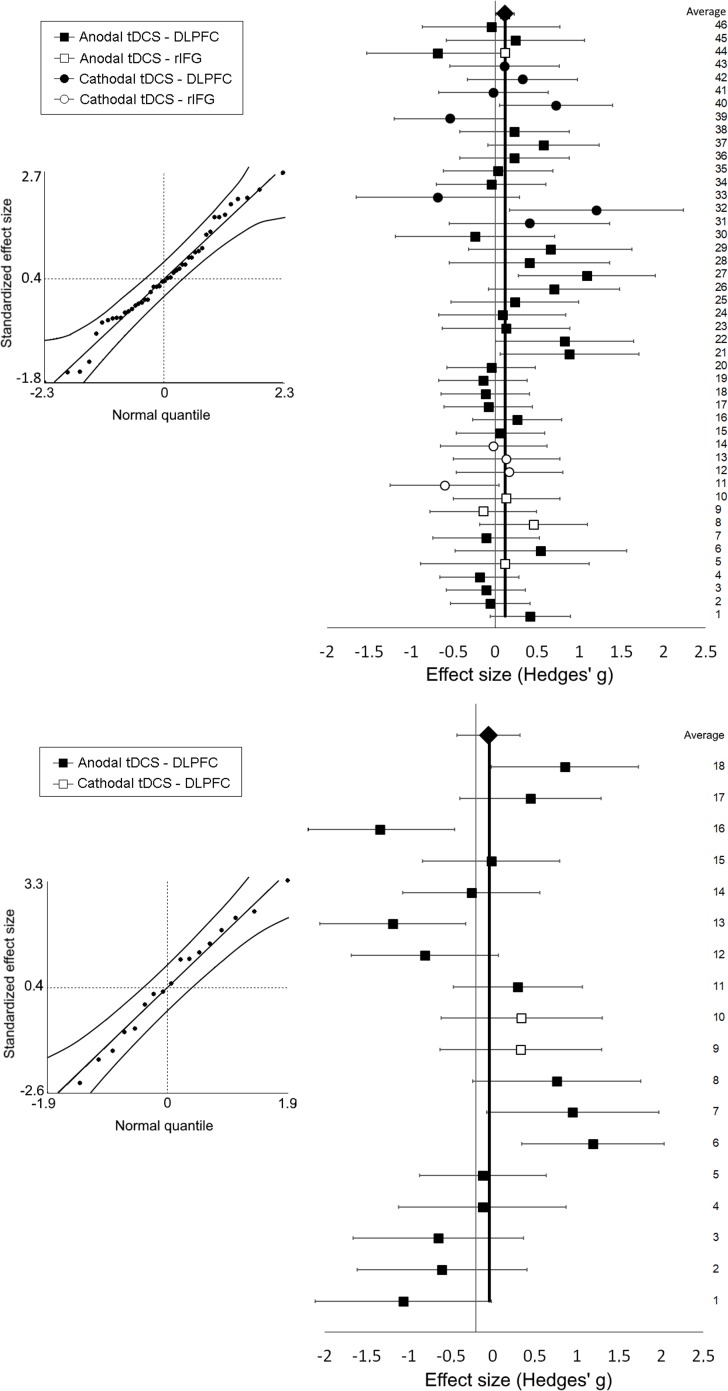
Meta-analysis and forest plot results including Hedges’ g and 95% confidence interval and Cumulative effect size of tDCS on inhibitory control (top) and working memory (down).

In the **Abstract**, there is modification to the seventh and eighth sentences. The correct sentences are: “Additionally, a significant improving effect of tDCS WM speed (but not accuracy) was found with a medium effect size. Overall, this meta-analysis supports a beneficial effect of tDCS on inhibitory control and WM in ADHD with a small effect size.”

In the “*Effects of tDCS on inhibitory control in ADHD patients*” subsection of the **Results**, there are several errors throughout. The corrected “Effects of tDCS on inhibitory control in ADHD patients” subsection is as follows:

“A significant cumulative effect size (Ē) of 0.117 (Z = 2.10, p = 0.035) was observed for a general tDCS effect on inhibitory control, taking polarity not into account Kolmogorov-Smirnov’s test of normality showed that the distribution of the effect sizes was not significantly different from a normal distribution (lower bound *p* = 0.20) and total heterogeneity of the effect sizes was not significant (Qtotal = 46.13, p = 0.425). The fail-safe number indicated that 79 unpublished null-findings would be required to render the effect non-significant. Exploration of montage showed that only dlPFC stimulation (l-dlPFC and bilateral) (Ē = 0.145, Z = 2.29, p = 0.021), but not rIFG stimulation (Ē = 0.005, Z = 0.04, p = 0.971) yielded a significant increase of accuracy rates in inhibitory control task performance.

Subsequently, polarity-dependent effects were investigated. Studies using anodal tDCS showed a significant Ē of 0.124 (Z = 2.13, p = 0.033), with a fail-safe number of 57 showing that anodal tDCS significantly improved inhibitory control. This sample was distributed normally (lower bound *p* = .20) and showed no significant heterogeneity (Qtotal = 33.07, p = 0.464). As for the stimulation polarity-independent analysis, this effect was driven by studies using a left and bilateral dlPFC montage (Ē = 0.133, Z = 2.07, p = 0.038), whereas the rIFG montage did not yield a significant effect (Ē = 0.084, Z = 0.33, p = 0.745). In contrast to anodal tDCS, cathodal tDCS did not show a significant overall effect (Ē = 0.073, Z = 0.47, p = 0.637).

Finally, an analysis was performed separating outcomes measures that focused on accuracy or amount of errors compared to the speed of response. The results showed no significant cumulative effect of tDCS on accurate responses in inhibitory control tasks (Ē = 0.113, Z = 1.51, p = 0.131). No significant cumulative effect was found for speed neither (Ē = 0.123, Z = 1.39, p = 0.164). For this last analysis, a deviation from normality was observed (p = 0.031). Results are summarized in Table 3.”

In the “*Effects of tDCS on working memory in ADHD patients*” subsection of the **Results**, there are several errors throughout. The corrected “Effects of tDCS on working memory in ADHD patients” subsection is as follows:

“No significant cumulative effect was observed for tDCS on working memory, without taking polarity into account (Ē = 0.150, Z = 0.78, p = 0.434). Also, no effect of tDCS was observed when only studies with an anodal montage were included (Ē = 0.103, Z = 0.48, p = 0.630). However, when separating outcomes for accuracy and speed, a significant effect of tDCS on speed was observed. TDCS led to a faster response time (Ē = 0.659, Z = 2.65, p = 0.008), with a fail-safe number of 16. The sample was normally distributed (lower bound *p* = .20) and no significant heterogeneity was seen (Qtotal = 5.88, p = 0.437). These results should be interpreted with caution, given the low sample size (N = 7). Moreover, results showed that tDCS did have no significant effect on accuracy of working memory task performance (Ē = -0.192, Z = -0.78, p = 0.433). Results are shown in Table 4.”

In the **Discussion**, there are errors in the second sentence of the second paragraph (fifth reported results). The correct sentence is: “Further sub-analyses yielded the following findings: (1) tDCS has an overall significant cumulative effect on inhibitory control in ADHD with a small effect size, (2) when the targeted brain region is taken into account, only tDCS over the dlPFC had a significant effect on inhibitory control (small effect size), but not tDCS over the rIFG, (3) when stimulation polarity was taken into account, only anodal, but not cathodal tDCS had a significant effect on inhibitory control, (4) when both polarity and targeted region are taken into account, only anodal tDCS of the dlPFC had a significant effect on inhibitory control with a small effect size, (5) and when analyzing inhibitory control outcomes separately, tDCS had no significant cumulative effect neither on accuracy, nor speed (i.e., reaction time).”

In the “tDCS effects on inhibitory control in ADHD” subsection of the Discussion, there is an error in the first sentence of the second paragraph. The correct sentence is: “Anodal dlPFC tDCS had the largest effect size (despite of small effect) on inhibitory control in ADHD populations, whereas anodal rIFG tDCS had no significant effect.”

The following sentence is missing from the **Conclusion** subsection: “However it is of note that all of the cumulative significant effect sizes were almost small except the one for effects of tDCS on working memory speed.” The corrected Conclusion subsection is as follows:

“The findings of this meta-analysis of tDCS interventions in ADHD suggest an improvement of neuropsychological deficits (i.e., inhibitory control and WM) by tDCS. Stimulation polarity and target area are relevant for the efficacy of tDCS in ADHD. Anodal dlPFC tDCS had a significantly superior effect on inhibitory control compared to cathodal/sham stimulation and anodal rIFG tDCS. TDCS significantly increased response accuracy of inhibitory control performance and decreased response time in WM tasks. However it is of note that all of the cumulative significant effect sizes were almost small except the one for effects of tDCS on working memory speed. Although our findings suggest improving effects of tDCS in ADHD neuropsychological deficits, the clinical utility of tDCS cannot be firmly rated with the currently available findings. Application of this method as a therapeutic intervention will require optimizing stimulation protocols based on general stimulation parameters and individual and inter-individual factors for improvement of clinical efficacy, exploration of clinical symptoms in addition to surrogate parameters, and achievement of sustained clinical benefits by tDCS over longer durations of time. Thus, future research is needed to more thoroughly explore and refine optimal stimulation parameters required for tDCS-based cognitive improvement and implementing robust experimental designs in different ADHD subtypes. Broadly speaking, the potential for tDCS as a non-invasive brain stimulation technique to safely improve neuroplasticity and treat neurological and neurodevelopmental disorders is encouraging. Future studies utilizing tDCS will further increase our understanding of neural networks and how to treat their pathological states in ADHD and other neurodevelopmental disorders including autism and learning disabilities.”
